# Prevalences and risk factors of overweight and obesity among kindergarten children in Shenzhen, China: a cross-sectional study

**DOI:** 10.3389/fpubh.2026.1780223

**Published:** 2026-03-11

**Authors:** Yuan-shuo Tian, Xue-yan Ma, Xiang-zheng Yang, Hong-zhi Yin, Yang Wang, Chao-jun Long, Xue-ying Qin, Chen Bai, Fei Dong, Zhen-dong Wang, Tie-gang Liu, Xiao-hong Gu

**Affiliations:** 1 Beijing University of Chinese Medicine, Beijing, China; 2 Third Affiliated Hospital, Guangzhou University of Chinese Medicine, Guangzhou, China; 3 Institute of Chinese Medicine Epidemic Disease, Beijing University of Chinese Medicine, Beijing, China; 4 Department of Pediatrics, Shenzhen Hospital (Longgang), Beijing University of Chinese Medicine, Shenzhen, China; 5 School of Traditional Chinese Medicine, Beijing University of Chinese Medicine, Beijing, China

**Keywords:** feeding behavior, kindergarten children, life habit, obesity, overweight

## Abstract

**Aim:**

Obesity in children have become major public health issues worldwide. The study aimed to determine the prevalence of overweight and obesity among children in Longgang District, Shenzhen, China, in the context of COVID-19 and analyze associated risk factors.

**Methods:**

A cross-sectional study was conducted through online survey from May to July 2021. World Health Organization child growth standards and the health industry standards of the People’s Republic of China were used as the diagnostic criteria for overweight and obesity. The prevalences of overweight and obesity were calculated, and risk factors of obesity were analyzed using binary logistic regression.

**Results:**

The parents or guardians of 124,593 children were administered, and 108,581 subjects were included. The prevalences of overweight and obesity among kindergarten children in Longgang District, Shenzhen, were 8.3 and 7.9%, respectively. The risk factors for childhood obesity included congenital, family, and lifestyle factors.

**Conclusion:**

In 2021, the obesity prevalence of kindergarten children in Longgang District was close to some high-income countries and higher than Shenzhen before the outbreaks of COVID-19. This finding may be associated with social, family, and personal factors. Exploration of evidence-based, effective means of modifying children’s health habits, and social support is urgently needed.

## Highlights

- In 2021, the prevalences of overweight and obesity among kindergarten children in Longgang District, Shenzhen, were 8.3 and 7.9%, respectively.
- This study provided epidemiological evidence of pediatric obesity among kindergarten children in Longgang District, Shenzhen, China, in 2021, while there are few large sample global or Chinese cross-sectional studies after 2020, especially during COVID-19.
- The risk factors for childhood obesity included congenital, family, and lifestyle factors.

## 1 Introduction

Obesity in children has become one of the world’s most pressing public health issues. There were 5.7% or 38.9 million overweight/obese children under five globally in 2020 (1). Being overweight and obese can affect a child’s quality of life and health throughout their lives. Childhood obesity increases the risk of lifelong obesity and is associated with cardiometabolic disease and cancer in adulthood (2). Childhood overweight/obesity and its adverse health consequences impose substantial burdens on the global healthcare system. The lifetime direct medical costs of obese children are an estimated $19,000 higher than that of normal-weight children (3). COVID-19-related control measures such as public places management and suspension of offline classes in kindergartens have changed children’s habits and customs, which might increase the prevalence of obesity among children.

## 2 Methods

### 2.1 Study design

A cross-sectional study was conducted using a census to identify children from kindergartens in Longgang District, Shenzhen. An online questionnaire collected demographic information, height, weight, birth data, dietary behavior, parental rearing behavior, and others. The prevalences of obesity and overweight were calculated, and the risk factors were analyzed using binary logistic regression. The questionnaire included questions according to kindergarten codes, which had been encoded before the survey to evaluate the quality of the data collected.

### 2.2 Population and diagnostic criteria

We investigated kindergarten children in the Longgang District of Shenzhen City using a general survey from May to July 2021. The researcher contacted the directors of all kindergartens in the district and explained the contents and purpose of the survey. If the kindergarten director agreed to participate in this survey, all children who met the selection criteria were included.

The inclusion criteria were as follows: children attended kindergartens in Longgang District, Shenzhen, in the second semester from 2020 to 2021; guardians with reading and writing skills were willing and able to complete the survey. Exclusion criteria were children who did not complete or submit the questionnaire to the online system.

Criteria for the diagnosis of overweight and obesity: Children under 60 months old were judged according to the WHO Child Growth Standards for children under 5 years (4). If the *Z*-score of weight-for-height (WHZ) was more than two but less than or equal to three, the child was considered overweight. If the WHZ was more than three, the child was considered obese. Five-year-old children (60–71.9 months old) were judged according to the WHO Growth reference data for 5–19 years (5). The child was considered overweight if the *Z*-score of body mass index (BMIZ) was more than one but less than or equal to two. If the BMIZ was more than two, the child was considered obese. Children aged six and above were judged according to the People’s Republic of China’s “screening for overweight and obesity among school-age children and adolescents” (WS/T 586–2018) (6). If the child’s body mass index was no more than the cutoff value, the child was judged overweight or obese.

### 2.3 Data collection

All investigators were trained uniformly, and only those who passed the examination participated in the investigation. We established standard operating procedures for the investigation to ensure the survey’s quality. The investigator introduced the survey to guardians of children through online and offline meeting and training video, explained the requirements for filling out the questionnaire in detail, and provided a unique identification code for the subject. The guardians could enter the online survey system with the code. The home page of the electronic survey provided the basic introduction and informed consent form. The subjects could voluntarily choose whether they would agree to participate.

Data included the following:

1) Demographic characteristics: gender, date of birth, ethnicity, only child or not.
2) Height and weight: We referred to the WHO “Physical status the use and interpretation of anthropometry” (7) to teach parents/guardians to measure height and weight to guardians. The height was in cm, and the weight was in kg, rounded to one decimal place.
3) Birth information: birth weight, gestational age, and birth mode.
4) Family information: parents’ educational qualifications, per capita family income, and the primary caregiver at home.
5) Lifestyle.

(i) Sleep duration

We referred to “sleep hygiene guidelines for children aged 0 to 5” (WS/T 579–2017) (8) and “management standards of compulsory education schools” (9) to determine whether children’s sleep time was sufficient. Adequate sleep for children under 3 years old is at least 11 h a day, and for children over 3 years old is at least 10 h a day.

(ii) Activity level

Referring to “Physical activity guideline for Chineses preschoolers aged 3–6 years” (10) and WHO “Guidelines on Physical Activity, Sedentary Behaviour and Sleep for Children under 5 Years of Age” (11), there were four activity levels based on children’s daily moderate-to-high-intensity physical activity time: basically inactive (time < 30 min), less active (30 min ≤ time <60 min), generally active (60 min ≤ time <90 min), and intensely active (time ≥90 min). Regarding the assessment of exercise intensity, common moderate-intensity activities for preschool children include brisk walking, ball throwing and catching, and jogging; high-intensity activities include sprinting (12). The assessment should also incorporate respiratory rat. The changes in children’s breathing and speech rate during exercise to provide a simple judgment of exercise intensity (13). For instance, during moderate-intensity exercise, children exhibit rapid breathing, meaning they can only speak short sentences and cannot express complete long sentences. In contrast, during high-intensity exercise, rapid and labored breathing occurs, making verbal communication impossible.

(iii) Eating behavior in the previous year

This category includes meal time (<25 min, 25–45 min, >45 min), eating less than their peers, eating slowly, not being interested in food, having a strong preference for certain textures or types of food (e.g., crispy, creamy, high-sugar, high-fat foods), irregular dining places, and inattention while eating. Children demonstrating this behavior more than 3 days a week were considered to have this dietary behavior. The questionnaire items assessing children’s eating behaviors were adapted from existing questionnaires and combines with published related researches. The questionnaires we referred to were the “Identification and Management of Feeding Difficulties” (14) and the “Chinese Preschoolers’ Eating Behavior Questionnaire” (15). Published research findings on dietary behavior surveys of Chinese children indicated that the main behavioral issues among preschool children in China are concentrated in picky eating, prolonged feeding time, and poor eating habits (16–19). Therefore, this survey primarily covers these aspects.

(iv) Feeding behavior of caregivers in the previous year

This category includes inducing children to eat (e.g., toys, television, story reward), allowing them to choose their food at will, and allowing them to snack freely. These behaviors were recorded if the caregiver engaged in such behavior more than 3 days per week. The questionnaire assessing the caregivers’ feeding practices were developed by referencing the classification of parenting styles. According to Baumrind’s taxonomy, parenting styles can be divided into three types: authoritarian, permissive and authoritative. Authoritarian has high requirements and low responsiveness, permissive has low requirements but high responsiveness, authoritative has both high requirements and responsiveness (20, 21). According to census data, Chinese family structure shows a significant trend toward miniaturization, leading to an increasing phenomenon of indulgent parenting, with a growing emphasis on the importance of family education (22–24), and a predominance of permissive parenting styles. Therefore, this study focused on investigating permissive feeding behaviors. The related items were referenced to the Child Feeding Questionnaire (25).

6) Antibiotic use in children within 1 year of age. The child’s caregivers (most of whom were the child’s mother) filled in all this information.

### 2.4 Statistical methods

Categorical data were expressed as percentages and 95% confidence intervals (CI). According to the data type, the chi-square test or Fisher’s exact test was used to compare the differences between groups. Binary logistic regression was used to calculate the correlations between obesity and demographic characteristics, birth status, children’s lifestyle, and caregivers’ feeding behavior in the previous year. The confounding factors (e.g., age, gender, ethnicity, and so on) were controlled for in the statistical analysis of risk factors. The statistical analysis was conducted using SPSS 20.0 (IBM, New York, USA). Two-sided *p*-values less than 0.05 were statistically significant.

### 2.5 Ethical approval and consent

This study was performed in line with the principles of the Declaration of Helsinki. The ethics review committee of Shenzhen Hospital of Beijing University of Traditional Chinese Medicine (Longgang) approved the study (12th April, 2021/No. SZLDH2021LSYM-030). The home page of the electronic survey provided the informed consent form. The subjects could voluntarily choose whether they would agree to participate, those who chose to agree to participate filled out the questionnaire.

## 3 Results

**Figure 1:**
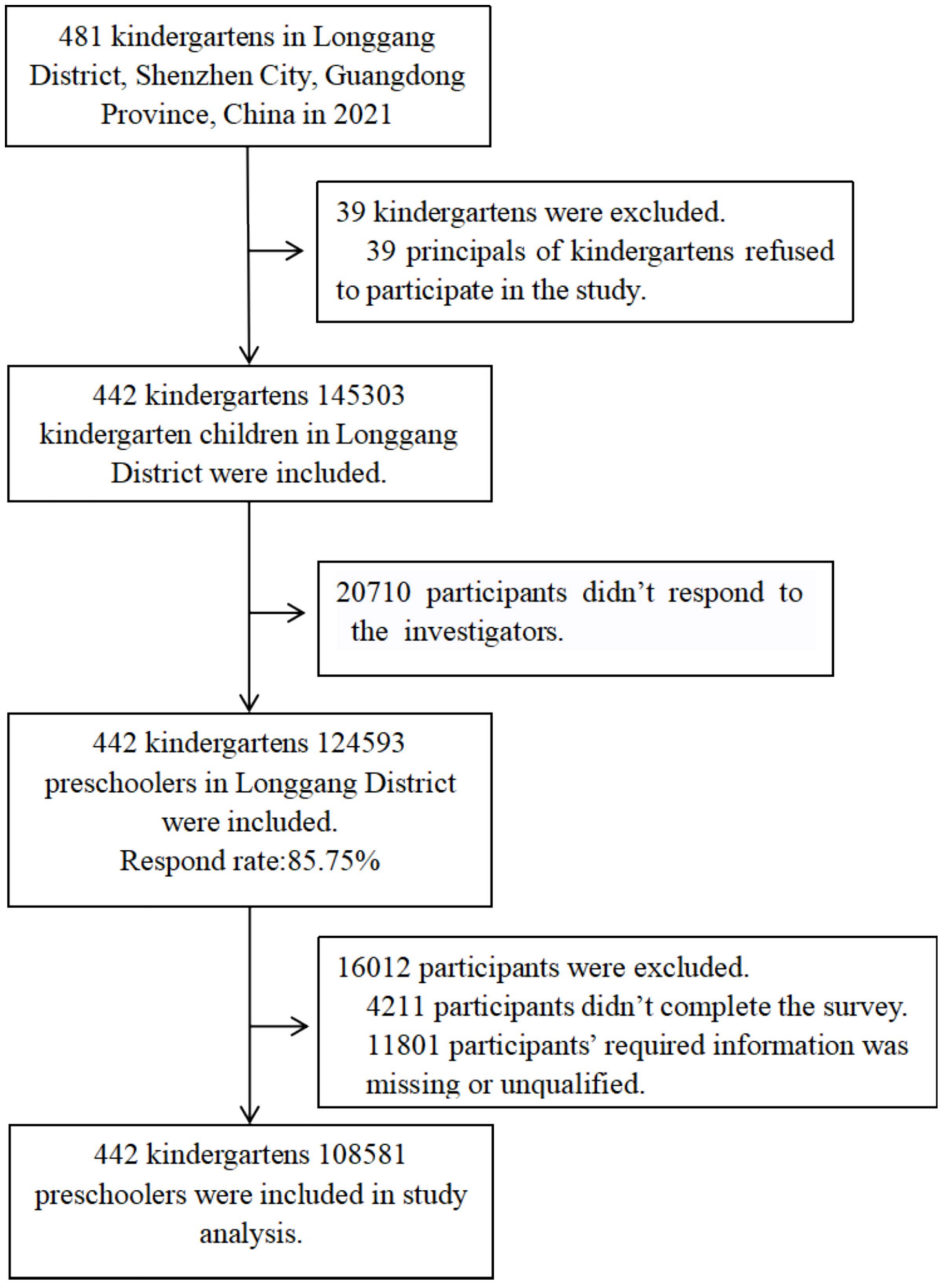
Flowchart of participants.

During the investigation, there were 481 kindergartens in Longgang District, Shenzhen; 39 (8.11%) kindergartens refused to participate in the survey, and 442 (91.89%) agreed. A total of 145,303 children from 442 kindergartens were investigated. There were 124,593 in the survey, for a response rate of 85.75%. There were 4,211 participants who failed to complete the survey, and 11,801 participants for whom critical information was missing, including birth date, height, or weight, or whose data were inconsistent with data quality requirements. Finally, 108,581 subjects were included for an effective rate of 87.15% (Figure 1). A total of 108,113 subjects answered the test item correctly (99.57%). Among them, there were 57,680 male children and 50,901 female children. The average age is 4.74 years (≤ 2 years: 134 children, 3 years: 8828 children, 7 years: 783 children, maximum age of 7 years).

The prevalence of overweight was 8.3% (95% CI 8.1 to 8.5%). The overweight prevalence of children under 6 years old was 6.5%. There were significant differences in overweight prevalence among ages, sexes, and parents’ educational qualifications. There were no significant differences between groups for ethnicities, per capita income of families, or primary caregivers (Table 1).

**Table 1:** Prevalence of overweight among kindergarten children in Longgang District, Shenzhen City, China in 2021(*n* = 108,581).

Characteristics	No. of participants	No. with overweight	Prevalence, % (95% CI)	*p*-value
Overall	108,581	9,024	8.3 (8.1 to 8.5)	
Age (years)				<0.01
Age<3	134	4	3.0 (0.1 to 5.9)	
3 ≤ age<6	80,890	5,269	6.5 (6.3 to 6.7)	
6 ≤ age	27,557	3,751	13.6 (13.2 to 14.0)	
Sex				<0.01
Male	57,680	5,121	8.9 (8.6 to 9.1)	
Female	50,901	3,903	7.7 (7.4 to 7.9)	
Ethnicity				0.164
Han nationality	103,494	8,628	8.3 (8.2 to 8.5)	
Other nationalities	5,087	396	7.8 (7.0 to 8.5)	
Education (father)				<0.01
Junior high school and below	18,667	1,635	8.8 (8.4 to 9.2)	
High school and junior college	51,261	4,370	8.5 (8.3 to 8.8)	
Bachelor’s degree or above	38,653	3,019	7.8 (7.5 to 8.1)	
Education (mother)				<0.01
Junior high school and below	20,192	1815	9.0 (8.6 to 9.4)	
High school and junior college	55,488	4,659	8.4 (8.2 to 8.6)	
Bachelor’s degree or above	32,901	2,550	7.8 (7.5 to 8.0)	
Per capita monthly income (¥)				0.097
Less than 5,000	23,745	1981	8.3 (8.0 to 8.7)	
5,000 ~ 10,000 (5,000 included)	35,383	2,990	8.5 (8.2 to 8.7)	
10,000 ~ 20,000 (10,000 included)	28,030	2,362	8.4 (8.1 to 8.8)	
No less than 20,000	12,955	1,047	8.1 (7.6 to 8.6)	
Not filled in	8,468	644	7.6 (7.0 to 8.2)	
The primary caregiver at home				0.156
Father/mother	93,251	7,699	8.3 (8.1 to 8.4)	
Ancestors (Grandpa/Grandma)	11,908	1,034	8.7 (8.2 to 9.2)	
Parents and grandparents	2,294	183	8.0 (6.9 to 9.1)	
Others (non-immediate family, Nanny)	1,128	108	9.6 (7.9 to 11.3)	

The prevalence of obesity was 7.9% (95% CI 7.7 to 8.1%). The obesity prevalence of children under 6 years old was 5.6%. Among obese children, the stunting prevalence was 12.4%. There were significant differences in the obesity prevalence among ages, sexes, parents’ educational qualifications, and per capita income of families. There were no significant differences between groups for ethnicities or primary caregivers of children (Table 2).

**Table 2:** Prevalence of obesity among kindergarten children in Longgang District, Shenzhen City, China in 2021 (*n* = 108,581).

Characteristics	No. of participants	No. with obesity	Prevalence, % (95% CI)	*p*-value
Overall	108,581	8,578	7.9 (7.7 to 8.1)	
Age (years)				<0.01
Age<3	134	2	1.5 (0.6 to 3.6)	
3 ≤ age<6	80,890	4,565	5.6 (5.5 to 5.8)	
6 ≤ age	27,557	4,011	14.6 (14.1 to 15.0)	
Sex				<0.01
Male	57,680	4,996	8.7 (8.4 to 8.9)	
Female	50,901	3,582	7.0 (6.8 to 7.3)	
Ethnicity				0.590
Han nationality	103,494	8,166	7.9 (7.7 to 8.1)	
Other nationalities	5,087	412	8.1 (7.3 to 8.8)	
Education (father)				<0.01
Junior high school and below	18,667	1922	10.3 (9.9 to 10.7)	
High school and junior college	51,261	4,161	8.1 (7.9 to 8.4)	
Bachelor’s degree or above	38,653	2,495	6.5 (6.2 to 6.7)	
Education (mother)				<0.01
Junior high school and below	20,192	2,108	10.4 (10.0 to 10.9)	
High school and junior college	55,488	4,383	7.9 (7.7 to 8.1)	
Bachelor’s degree or above	32,901	2087	6.3 (6.1 to 6.6)	
Per capita monthly income (¥)				<0.01
Less than 5,000	23,745	2,160	9.1 (8.7 to 9.5)	
5,000–10,000 (5,000 included)	35,383	2,795	7.9 (7.6 to 8.2)	
10,000–20,000 (10,000 included)	28,030	2067	7.4 (7.1 to 7.7)	
No less than 20,000	12,955	924	7.1 (6.7 to 7.6)	
Not filled in	8,468	632	7.5 (6.9 to 8.0)	
The primary caregiver at home				0.079
Father/mother	93,251	7,299	7.8 (7.7 to 8.0)	
Ancestors (Grandpa/Grandma)	11,908	1,011	8.5 (8.0 to 9.0)	
Parents and grandparents	2,294	175	7.6 (6.5 to 8.7)	
Others (non-immediate family, Nanny)	1,128	93	8.2 (6.6 to 9.9)	

Binary multivariate logistic regression showed that risk factors for obesity were male sex (odds ratio OR = 1.20), older age (OR = 4.49, 13.19), high birth weight (OR = 1.43), cesarean delivery (OR = 1.08), parents with low education levels (father: OR = 1.13, 1.22, mother: OR = 1.12, 1.30), family per capita income less than 5,000¥ (OR = 1.08), maternal childbearing age less than 20 (OR = 1.21), accompanied by grandparents alone (OR = 1.33), non-only-child (OR = 1.12), inadequate physical activity (OR = 1.24, 2.10), insufficient sleep duration (OR = 1.15), short or long meal times (OR = 1.41, 1.09), strong preference for certain textures or types of food (OR = 1.08), irregular dining places (OR = 1.11), and allowing children to snack freely (OR = 1.22) (Table 3).

**Table 3:** Logistic regression models for obesity among kindergarten children in Longgang District, Shenzhen City, China in 2021 (*n* = 108,581).

Characteristics	Adjusted odds ratio (95% CI)^*^	*p-*value
Congenital factors		
Sex (reference: Female)		
Male	1.202 (1.165 to 1.240)	<0.001
Age (reference: age < 3 years)		
3 years ≤ age < 6 years	4.494 (1.683 to 11.999)	0.003
6 years ≤ age	13.189 (4.939 to 35.220)	<0.001
Birth weight (reference: ≤4 kg)		
High birth weight (>4 kg)	1.430 (1.341 to 1.525)	<0.001
Maternal delivery (reference: natural labor)		
Cesarean delivery	1.078 (1.044 to 1.113)	<0.001
Not filled in	2.134 (1.129 to 4.037)	0.020
Family factors		
Father’s education (reference: bachelor’s degree or above)		
High school and junior college	1.126 (1.081 to 1.173)	<0.001
Junior high school and below	1.218 (1.150 to 1.290)	<0.001
Mother’s education (reference: bachelor’s degree or above)		
High school and junior college	1.120 (1.073 to 1.169)	<0.001
Junior high school and below	1.303 (1.228 to 1.382)	<0.001
Per capita monthly income of the family (¥) (reference: ≥20,000)		
10,000–20,000 (10,000 included)	1.017 (0.962 to 1.075)	0.557
5,000–10,000 (5,000 included)	1.025 (0.970 to 1.082)	0.387
Less than 5,000	1.082 (1.020 to 1.147)	0.009
Not filled in	0.932 (0.859 to 1.011)	0.089
Maternal childbearing age (reference: 20-35years)		
Age < 20 years	1.214 (1.045 to 1.412)	0.011
Age > 35 years	0.980 (0.928 to 1.035)	0.466
Not filled in	1.230 (1.125 to 1.345)	<0.001
The primary caregiver at home (reference: parents)		
Ancestors (Grandpa/Grandma)	1.330 (1.267 to 1.396)	<0.001
Parents and grandparents	1.087 (0.976 to 1.210)	0.130
Others (non-immediate family, Nanny)	1.031 (0.890 to 1.195)	0.681
Non-only-child	1.120 (1.079 to 1.162)	<0.001
Lifestyle factors		
Physical activities (reference: more intense activity)		
General activity	0.906 (0.878 to 0.936)	<0.001
Less activity	1.240 (1.148 to 1.340)	<0.001
Basically inactive	2.101 (1.266 to 3.485)	0.004
Not filled in	1.046 (0.930 to 1.176)	0.452
Sleep time (reference: sufficient)		
Not enough	1.149 (1.114 to 1.186)	<0.001
Not filled in	1.300 (0.639 to 2.645)	0.470
Meal time (reference: 25–45min)		
<25 min	1.411 (1.366 to 1.457)	<0.001
>45 min	1.085 (1.010 to 1.165)	0.025
Children eating behavior		
Eating less than their peers	0.738 (0.696 to 0.783)	<0.001
Eating slower than their peers	0.694 (0.660 to 0.730)	<0.001
Strong preference for certain textures or types of food	1.078 (1.033 to 1.125)	<0.001
An irregular dining place	1.114 (1.039 to 1.194)	0.003
Feeding behavior of caregivers		
Inducing children to eat (toys, television, story reward)	0.930 (0.867 to 0.998)	0.045
Allowing children to choose their food at will	0.928 (0.887 to 0.971)	0.001
Allowing children to snack freely	1.218 (1.130 to 1.312)	<0.001
Antibiotic use within 1 year old (reference: unused)		
Used	0.777 (0.728 to 0.828)	<0.001
Not filled in	0.935 (0.898 to 0.974)	0.001

^*^Adjusted for all characteristic variables in table.

## 4 Discussion

Our main finding was that the prevalence of overweight among kindergarten children was 8.3% (8.1–8.5%), and the prevalence of obesity was 7.9% (7.7–8.1%) in Longgang District, Shenzhen, in 2021. The overweight and obesity prevalences of children under 5 years old was 7.1%, higher than the worldwide prevalence in 2020 (5.7%), lower than in North America (9.1%) and Australia/New Zealand (16.9%), equivalent to that of Western Europe (7.0%), and higher than that of Southeast Asia (3.3%) (1). This study showed that in Longgang District, Shenzhen, the overweight prevalence of children under 6 years old was 6.5%, and the obesity prevalence was 5.6%. The overweight prevalence was slightly lower than the national prevalence (6.8%) and urban prevalence (6.9%) in China, and the obesity prevalence is higher than the national (3.6%) and urban prevalence (3.4%) (26).

Childhood obesity positively correlates with economic development (27). Shenzhen is a Special Economic Zone enjoying a golden development period in China. In 2017–2021, the gross domestic product of Shenzhen ranked third in China, while the market economy and living standards improved rapidly. Longgang District is located in the northeast of Shenzhen. It has developed rapidly in the electronic information, new energy and other emerging industries, and has been ranked the first in the national top 100 industrial districts for seven consecutive years. In addition, Longgang District is a major area in terms of population, industry and innovation, with a large number of migrant and floating population (28). In Shenzhen, a fast-paced city, people tend to choose fast food and processed foods, and have the habit of ordering at night. During the survey period, its food delivery orders ranked first nationwide (29). This study showed that the obesity prevalence of the investigated district was 7.9% in 2021, possibly illustrating the epidemiology of childhood obesity during COVID-19. Compared with a study in 2015, which showed that the obesity prevalence among kindergarten children was 5.96% in Shenzhen and 7.79% in the Longgang District (30), this study’s obesity prevalence was slightly higher than the prevalence before COVID-19. A Cohort Analysis in china found that the obesity prevalence slightly increased from 12.29% (2017) to 13.28% (2019) but substantially increased to 15.29% in 2020. The mean yearly change in BMI *Z*-score before and during the pandemic were 0.039 (95% CI = 0.037, 0.042) and 0.131 (95% CI = 0.125, 0.138), respectively, yielding a difference of 0.092 (95% CI = 0.087, 0.096). Similarly, changes and age- and sex-adjusted BMI increased by 0.191 (95% CI = 0.179, 0.202) during the pandemic compared with those of previous years (31). During the outbreak, children were requested to isolate at home and learn online to avoid gathering at kindergartens. Their way of life imposed substantial changes (32); for example, there were sudden decreases in outdoor activities but increased screen time. Unlike regular and quantitative feeding in kindergartens, eating and feeding on-demand and increased snack intake are more likely at home (33). Similarly, the daily routines disrupted by the pandemic also include sleep (34). Additionally, a systematic review reported that unhealthy eating habits, excessive behavioral stress, depression, anxiety, low mood also have been identified as risk factors for obesity during the COVID-19 pandemic (35). These are all possible reasons for the increased obesity prevalence of children during the epidemic.

We analyzed risk factors related to childhood obesity using binary logistic regression and identified congenital, family, and lifestyle factors. We found that the obesity prevalence of boys was 1.21 times that of girls, consistent with the results of several studies (30, 36). This finding is probably due to gender differences in childhood growth regularity (9). Higher age in this study correlated with a higher risk of childhood obesity. Studies found that 5 to 7 years old was a high incidence stage of childhood obesity (37). These findings suggest that adverse living habits as a child matures might cause adipose tissue accumulation. Obesity associated with stunting also might be the explanation, in the present study, the stunting prevalence among obese children was 12.4%, much higher than the prevalence among kindergarten children in Shenzhen Longgang District (3.3%) (38). This study showed that high birth weight (>4 kg) and cesarean section were risk factors for childhood obesity. Cesarean delivery significantly increases the risk of childhood obesity (37), possibly related to microbial colonization during birth (39).

The preschool years are critical for childhood obesity. It is essential to teach childhood nutrition and scientific feeding knowledge. The mother plays a critical role in their children’s development. Nutritional balance during pregnancy and natural delivery, where possible, are recommended. Except in cases where necessary for normal fetal development, excessive supplement is not necessary.

Parents with less education was a risk factor, consistent with the results of a case–control study in China (40). In the present study, family per capita income less than 5,000¥ was a risk factor for childhood obesity, a different result from that of the Chinese (1) but consistent with results from developed countries (41). Our finding suggests that, as living standards improve, Chinese people can access sufficient food sources regardless of income; and unhealthy food is even cheaper and more accessible. However, low-income families may lack scientific child-feeding knowledge.

We found that maternal childbearing age of less than 20 was a risk factor for childhood obesity. A study found that there might be a correlation between parents’ childbearing age and children who are overweight/obese (42). This present study found that, compared with parents as the primary caregiver at home, grandparents as caregivers correlated with a higher risk in obesity of children. In China, many grandparents are raising children, and grandparents’ traditional parenting concepts likely negatively impact children’s customs and growth (43). In the present study, not being an only child was a risk factor, which accords with another Chinese study (37). That study showed that a second child had a higher risk of obesity than the first. We suggest that society and schools should deliver health education about child-rearing for parents with low levels of education, low-income families, and grandparents. Against the background of policies that encourage childbearing, non-only child status, and health status deserve attention. Women and their partners should be provided effective guidance and encouragement on the appropriate ages for childbearing and informed of the risks.

Activity is a risk factor for childhood obesity. Lack of physical activity in early years had a negative influence on physical and cognitive development for children (44), but it was common in preschool children, because of home quarantine during COVID-19 (32). The present study found that lack of sleep increased the risk of obesity. A cohort study found that short sleep time significantly and independently increased children’s probability of being overweight (45).

Poor diet and feeding behaviors are closely associated with overweight and obesity in children (46). Too short or too long mealtimes are not good eating behavior, possibly related to a strong appetite causing excessive energy intake and obesity (47). High-calorie and processed foods are standard in the beverage market, and children prefer sweets, fried food, and snacks. A study showed that children with “processing/snacks” dietary patterns had higher prevalences of obesity (48).

Quarantine increased food and snack consumption (33), promoting weight gain. In the present study, some statistically negative correlations for childhood obesity include eating less and more slowly than their peers, inducing children to eat (e.g., toys, television, and stories rewards), and allowing children to choose their food at will. There are different views on inducing children to eat this feeding behavior and its effect on children’s eating behavior. Some scholars have proposed that (49, 50), although inducing children to eat may be short-term effect, but the child is taught to eat for external reasons. The food intake is regulated by external and not internal factors (hunger, satiety), which may increase risk of eating disorders, affect children’s growth and development. But some scholars have proposed that, the initial level of liking for food may lead to different outcomes. Declines in preference following a reward contingency have typically been observed with reasonably well-liked foods (51). Rewarding children to eat may promote their consumption of foods they dislike, with no negative effects on liking (52), which can promote a more balanced dietary structure. A large study demonstrated that rewarding children for tasting an initially-disliked food produced sustained increases in acceptance (52). We suggest that parents guide children appropriately regarding increased exercise, especially indoor activity during COVID-19. Increasing sleep duration might reduce childhood obesity. It is also essential to develop good eating habits, e.g., moderate eating speed, proper feeding, and reducing snacks. Allowing children to choose their food at will might improve their eating autonomy.

In the present study, antibiotic use within 1 year of age was a statistically negative correlation for childhood obesity. This finding aligns with the results of Uzan-Yulzari et al. (53), who reported that boys receiving antibiotic treatment after birth exhibit slower growth in weight and height during early life. However, more studies suggest that early-life antibiotic use increases the risk of obesity (54, 55). The impact of antibiotics on childhood weight may be related to factors such as the type of antibiotic, duration of treatment, and time of exposure, as well as confounding factors including the reason for antibiotic use (empirical antibiotic therapy for suspected infections, infectious diseases), and perinatal antibiotic therapy (56, 57). Future studies should incorporate these factors to further clarify the effects of early-life antibiotic use on childhood weight.

## 5 Study strengths and limitations

The strengths of this study are as follows. First, the census format and large sample size allowed us to obtain comprehensive data and explore risk factors associated with childhood obesity. Second, we used an online questionnaire, which was more convenient and economical than the traditional paper questionnaire. During home quarantine, online questionnaires have better operability and safety. Finally, the study had a high response rate and good data integrity (i.e., questionnaire efficiency).

This study has some limitations. First, there is the lack of direct measurement of height and weight in children. Heights and weights of children were collected by their parents. Determining overweight and obesity only on the basis of data from the questionnaire is very imprecise. Most studies state that there is a difference between self-reported data and measured data. The self-reported data may lead to overestimating the prevalence of overweight and obesity among children (58). Others believe that self-reported weight and height are sufficient to classify overweight/obesity correctly (59). We explained the measuring methods of height and weight to parents in detail, and the correct rate of the questionnaire test was satisfactory (99.55%), reflecting the accuracy of the survey result. Nevertheless, we believe that bias caused by self-reported data should not be ignored. Second, in this study, the majority of parents were aged 25–39. We evaluated the educational characteristics of participating families by considering the educational attainment of this age group in Shenzhen. According to the 2020 Shenzhen Population Census Yearbook (60), the distribution of education among Shenzhen residents aged 25–39 shows: with junior high school or below (33.05%), with high school and junior college (43.31%), with bachelor’s degree or above (23.64%). The study revealed that the distribution of education among participating parents was as follows: junior high school or below (17.19%), high school and junior college (47.21%), and bachelor’s degree or above (30.30%). Therefore, it is inferred that families with higher education are more likely to participate in this survey. However low-educated families are significant risk factors for children overweight and obesity, so this survey may underestimate the prevalence of children overweight and obesity, which may lead to underestimating the prevalence in this study. Third, parental BMI is a important predictor of childhood obesity, and it was not collected in the analysis because of the study design constraints. The study focused on the correlation between children’s lifestyle, family environment, and childhood obesity; other key relevant factors should be included in the future.

## 6 Conclusion

In 2021, the overweight and obesity prevalences of kindergarten children in Longgang District, Shenzhen, was close to that of high-income countries in Western Europe, and it was higher than the prevalence before COVID-19, which may be linked to social, family, and personal factors. Early childhood is a critical period for behavior formation and strongly depends on correct guidance by schools and families. Our findings suggest that parents play an irreplaceable role in preventing childhood obesity and promoting children’s health. They should master scientific childcare knowledge and guide children correctly. From a policy perspective, exploring scientific and practical ways and developing public health services to promote healthy behavior habits are essential.

## Data Availability

The raw data supporting the conclusions of this article will be made available by the authors, without undue reservation.
